# A prognostic score for non-small cell lung cancer resected after neoadjuvant therapy in comparison with the tumor-node-metastases classification and major pathological response

**DOI:** 10.1038/s41379-021-00777-y

**Published:** 2021-03-13

**Authors:** Philipp Zens, Corina Bello, Amina Scherz, Julia Koenigsdorf, Alexander Pöllinger, Ralph A. Schmid, Adrian Ochsenbein, Christina Neppl, Rupert Langer, Sabina Berezowska

**Affiliations:** 1 Institute of Pathology, University of Bern, Bern, Switzerland; 2 Department of Medical Oncology, Inselspital University Hospital Bern, Bern, Switzerland; 3 Department of Diagnostic, Interventional and Pediatric Radiology, Inselspital University Hospital Bern, Bern, Switzerland; 4 Department of General Thoracic Surgery, Inselspital University Hospital Bern, Bern, Switzerland; 5 Institute of Pathology, Kepler University Hospital and Johannes Kepler University, Linz, Austria; 6 Department of Laboratory Medicine and Pathology, Institute of Pathology, Lausanne University Hospital and University of Lausanne, Lausanne, Switzerland

**Keywords:** Non-small-cell lung cancer, Outcomes research

## Abstract

Studies validating the prognostic accuracy of the tumor-node-metastases (TNM) classification in patients with lung cancer treated by neoadjuvant therapy are scarce. Tumor regression, particularly major pathological response (MPR), is an acknowledged prognostic factor in this setting. We aimed to validate a novel combined prognostic score. This retrospective single-center study was conducted on 117 consecutive patients with non-small cell lung cancer resected after neoadjuvant treatment at a Swiss University Cancer Center between 2000 and 2016. All cases were clinicopathologically re-evaluated. We assessed the prognostic performance of a novel prognostic score (PRSC) combining T-category, lymph node status, and MPR, in comparison with the eighth edition of the TNM classification (TNM8), the size adapted TNM8 as proposed by the International Association for the Study of Lung Cancer (IASLC) and MPR alone. The isolated ypT-category and the combined TNM8 stages accurately differentiated overall survival (OS, stage *p* = 0.004) and disease-free survival (DFS, stage *p* = 0.018). Tumor regression had a prognostic impact. Optimal cut-offs for MPR emerged as 65% for adenocarcinoma and 10% for non-adenocarcinoma and were statistically significant for survival (OS *p* = 0.006, DFS *p* < 0.001). The PRSC differentiated between three prognostic groups (OS and DFS *p* < 0.001), and was superior compared to the stratification using MPR alone or the TNM8 systems, visualized by lower Akaike (AIC) and Bayesian information criterion (BIC) values. In the multivariate analyses, stage III tumors (HR 4.956, *p* = 0.003), tumors without MPR (HR 2.432, *p* = 0.015), and PRSC high-risk tumors (HR 5.692, *p* < 0.001) had significantly increased risks of occurring death. In conclusion, we support 65% as the optimal cut-off for MPR in adenocarcinomas. TNM8 and MPR were comparable regarding their prognostic significance. The novel prognostic score performed distinctly better regarding OS and DFS.

## Introduction

Lung cancer is one of the most frequent cancers worldwide and the leading cause of cancer-related death [1]. Approximately 80% of lung cancer patients are diagnosed with non-small cell lung cancer (NSCLC). One-third of them present at a locally advanced stage. This is a heterogeneous group for which treatment modalities encompass multimodal approaches [2]. In resectable locally advanced NSCLC, neoadjuvant chemotherapy is an important strategy, despite the lack of a clear benefit over adjuvant chemotherapy with respect to overall survival (OS) [3]. However, it is beneficial regarding three major points, comprising (a) consistent chemotherapy delivery for patients potentially not fit enough for adjuvant chemotherapy after surgery, (b) biological stress test for the tumor, and (c) avoidance of futile surgery [4]. Currently, there are no uniformly defined standards for neoadjuvant chemotherapy but it usually consists of platinum-based chemotherapy administered in four cycles [5]. Recently and based on the success of immunotherapy in the palliative setting, several studies tested antibodies blocking the programmed cell death protein 1 (PD1)/programmed cell death 1 ligand 1 (PD-L1) axis in the neoadjuvant setting, either as monotherapy or in combination with ipilimumab or chemotherapy. Especially the combination therapies reached promising major pathological response (MPR) rates of up to 85% [6]. In contrast, the use of tyrosine kinase inhibitors (TKI) and molecular-guided perioperative treatment is less clear and requires further investigation. For example, response rates to EGFR-targeting TKIs in the neoadjuvant setting seem lower than in patients with the advanced-stage disease [7]. Even though additional radiotherapy seems to improve local tumor control, long-term survival is not altered presumably because distant micrometastases are not reached [3].

Different prognostic models have been developed for locally advanced NSCLC, based on clinical and pathological parameters [8, 9]. However, only a limited number of patients receiving neoadjuvant treatment were included [8–10]. To our knowledge, there are no models explicitly designed for NSCLC resected after neoadjuvant treatment, though different clinicopathological parameters have been shown to significantly impact survival [11–13]. The amount of residual tumor is one important parameter [3, 14, 15]. Junker et al. described a favorable prognosis for patients with low percentages of residual tumor and the best prognosis in patients with a complete pathological response (pCR), i.e. no remnants of tumor cells in the resected tissue [14]. More recently, Patear et al. confirmed these results and proposed MPR, defined as ≤10% residual tumor cells, as a more practical marker due to only limited numbers of cases achieving pCR [15]. As for other solid tumors, tumor regression could be used as a surrogate marker in neoadjuvant studies and should be included in pathological reports [3, 16, 17].

In general, the UICC/AJCC tumor-node-metastases (TNM) classification remains the gold standard to predict cancer prognosis, but the dataset informing the current edition has explicitly excluded cases resected after neoadjuvant therapy, as have most validation studies [18–22]. The prognosis of patients with locally advanced NSCLC is dismal and the benefit from neoadjuvant chemotherapy is limited with only 5% benefit on 5-year OS [23]. In order to test and advance new therapeutic strategies in a more selective way, it is crucial to more accurately predict survival after neoadjuvant therapy using a practical approach.

In this study, we aimed to validate the current eighth edition of the UICC/AJCC TNM classification (TNM8) [18] juxtaposed with regression grading in a cohort of NSCLC resected after a neoadjuvant chemo- or radio-chemotherapy. We investigated the benefit of adjusting the tumor size informing the T-category as advocated by the IASLC and additionally propose a prognostic score with better stratification combining T-category, lymph node status, and tumor regression [16].

## Patients and methods

### Patient cohort

This retrospective single-center study was conducted on a cohort of consecutive patients with non-neuroendocrine NSCLCs after neoadjuvant treatment resected between January 2000 and December 2016 in the department of general thoracic surgery of the Inselspital Bern and diagnosed at the Institute of Pathology, University of Bern. The cohort was assembled using pathology files and was subsequently validated using the clinical files of the Inselspital Bern, the survival data by the cantonal cancer registry, and contacting general practitioners [24, 25].

This study was conducted according to the REMARK-criteria and approved by the Cantonal Ethics Commission of the Canton of Bern (KEK 2017-00830), which waived the requirement for written informed consent [26].

To guarantee neoadjuvant intention, only patients who had received at least 1 cycle of platinum-based chemotherapy were eligible for inclusion. Unclear cases were re-evaluated by consulting an experienced oncologist (AS). Initially, the cohort comprised 129 cases. After re-evaluation of the clinical files, ten patients were excluded due to lack of neoadjuvant intention of the therapy prior to resection and two patients due to missing material for appropriate evaluation of tumor regression. Finally, 117 cases were included in the study cohort (Supplementary Fig. S-1). There were 85 (73%) men and 32 (27%) women. The median age at surgery was 62 years (IQR 56–69). Detailed baseline characteristics are presented in Table 1. The neoadjuvant regimen was further grouped corresponding to the current standards at the Inselspital Bern. Hence, 64/117 (54.7%) patients received the standard regimen consisting of at least three cycles of platinum-based chemotherapy and an additional taxane and 53/117 (45.3%) received one cycle of platinum-based chemotherapy in combination with another cytotoxic agent or less than three cycles. The applied drug combinations are summarized in Table 1. In two patients the specific regimen was not reported but neoadjuvant treatment was confirmed by pathological or clinical documentation. An adaptation of chemotherapy was reported for 28/117 (23.9%) patients as adjustment of dose in 12 and change of drug in 16 cases. Most patients (*n* = 85) received three cycles of neoadjuvant chemotherapy and the neoadjuvant regimen was given over a median of 63 [IQR 62–80.5] days. One patient received a maximum of 12 cycles over a period of 256 days due to a change of therapy after bronchospasms. Two patients had to stop the neoadjuvant therapy after one cycle due to severe medical conditions (purulent effusion, myocardial infarction). The tumors were resected after a median of 29 [IQR 15–48] days. One patient received perioperative therapy and one patient was operated on with a delay of 222 days due to discontinuation of therapy during the fourth cycle and the following rehabilitation after pneumonia with subsequent worsening of the general condition. Adjuvant radio-chemotherapy was reported in four patients, isolated adjuvant chemotherapy in five patients, and isolated adjuvant radiotherapy for 16 patients.

**Table 1:** Baseline characteristics of the study cohort according to squamous cell carcinoma and adenocarcinoma histology.

	SQCC	ADC	*p*-Value	Other
	(*n* = 54)	(*n* = 56)		(*n* = 7)
Age, years (median [IQR])	63[55.25–69.75]	62[55.75–68.25]	0.788^a^	68[59–71]
Smoking status	*n* = 48 (%)	*n* = 49 (%)	0.193^b^	*n* = 6 (%)
Non-smoker	3(6.2)	9(18.4)		2(33.3)
Ex-smoker	27(37.5)	22(44.9)		4(66.7)
Active smoker	18(56.2)	18(36.7)		
Macroscopic tumor bed, cm (median [IQR])	3.5[2.58–5.42]	3.5[2.38–4.20]	0.385^a^	4[1.9–4.35]
Adjusted tumor size, cm (median [IQR])	1.24[0.06–2.76]	1.78[0.05–2.95]	0.832^a^	0[0–3.16]
ypT-TNM8	*n* = 54 (%)	*n* = 56 (%)	0.805^a^	*n* = 7 (%)
ypT0	6(11.1)	5(8.9)		4(57.1)
ypT1	14(25.9)	19(33.9)		
ypT2a	11(20.4)	9(16.1)		
ypT2b	3(5.6)	3(5.4)		1(14.3)
ypT3	6(11.1)	13(23.2)		1(14.3)
ypT4	14(25.9)	7(12.5)		1(14.3)
TNM8	*n* = 54 (%)	*n* = 56 (%)	0.557^a^	*n* = 7 (%)
No vital tumor	3(5.6)	3(5.4)		4(57.1)
Stage I	10(18.5)	12(21.4)		
Stage II	14(25.9)	11(19.7)		
Stage III	26(48.1)	26(46.4)		3(42.9)
Stage IV	1(1.9)	4(7.1)		
Regression	*n* = 54 (%)	*n* = 56 (%)	0.460^a^	*n* = 7 (%)
pCR	3(5.6)	4(7.1)		4(57.1)
<1%	9(16.7)	9(16.1)		
1–10%	9(16.7)	6(10.7)		
11–49%	9(16.7)	6(10.7)		1(14.3)
≥50%	24(44.3)	31(55.4)		2(28.6)
PRSC	*n* = 54 (%)	*n* = 56 (%)	0.332^a^	*n* = 7 (%)
Low risk	23(42.6)	21(37.5)		4(57.1)
Intermediate risk	14(25.9)	25(44.6)		1(14.3)
High risk	17(31.5)	10(17.9)		2(28.6)
Neoadjuvant chemotherapy	*n* = 53 (%)	*n* = 55 (%)	0.551^b^	*n* = 7 (%)
Cisplatin + docetaxel	31(58.5)	31(56.4)		6(85.7)
Carboplatin + paclitaxel	3(5.7)	2(3.6)		
Cisplatin + pemetrexed	6(11.3)	7(12.7)		
Cisplatin + gemcitabin	6(11.3)	3(5.5)		
Cisplatin + vinorelbin	2(3.8)	7(12.7)		1(14.3)
Cisplatin + etoposid		1(1.8)		
Other	5(9.4)	4(7.3)		
Neoadjuvant radiotherapy	*n* = 50 (%)	*n* = 54 (%)	0.689^b^	*n* = 7 (%)
No	38(76)	38(70.4)		4(57.1)
Yes	12(24)	16(29.6)		3(42.9)
Surgical procedure	*n* = 54 (%)	*n* = 56 (%)	0.001^b^	*n* = 7 (%)
Lobectomy	23(42.6)	42(75)		4(57.1)
Bilobectomy	4(7.4)	1(1.8)		
Pneumonectomy	27(50)	13(23.2)		3(42.9)
*R*	*n* = 53 (%)	*n* = 55 (%)	0.031^b^	*n* = 7 (%)
R0	41(77.4)	51(92.7)		7(100)
R1	12(22.6)	4(7.3)		

*SQCC* squamous cell carcinoma, *ADC* adenocarcinoma, *ypT-TNM8* ypT according TNM eighth edition, *TNM8* stages according TNM eighth edition, *pCR* pathological complete response, *PRSC* prognostic score.

^a^Mann–Whitney-*U* test.

^b^Fisher’s exact test.

Patients who were lost to follow up or died within 30 days after surgery were excluded from survival analyses. The disease-free survival (DFS) was defined as the interval between the start of treatment and loco-regional or metastatic recurrence or death from any cause. Relapse was defined as the clinical diagnosis of tumor recurrence ≥3 months after the resection. OS was defined as the time elapsed from the start of treatment to death of any cause. The start of treatment was defined as the start of neoadjuvant therapy. For three cases the date of resection was chosen for survival analyses as the dates of the neoadjuvant chemotherapy were not available but neoadjuvant treatment was explicitly mentioned in the pathological or clinical documentation. Survival data for analyses was restricted to 5 years after surgery due to the multimorbidity of patients. Survival analyses were possible for 105 patients after the exclusion of two patients due to missing survival information and ten patients due to the last follow-up date within 30 days after surgery. Further five patients were excluded due to distant metastases. Median OS was 43 months (95% CI = 32–NA) and 47 events occurred (Supplementary Fig. S-2). Median DFS was 21 months (95% CI = 15–29 months) and 68 events occurred (Supplementary Fig. S-2).

### Histological tumor types

Histological tumor type was re-evaluated for each case by two investigators (SB, PZ) according to the current 2015 World Health Organization criteria [27]. The cohort consisted of 54 (46.1%) squamous cell carcinomas (SQCC), 56 (47.9%) adenocarcinomas, 3 (2.6%) adenosquamous carcinomas, and 4 (3.4%) NSCLC not otherwise specified. The unspecified NSCLC showed pCR in the resection specimen, without residual tumor cells for immunohistochemical work-up, and no defined histology in the pathological report of the biopsies. One patient was diagnosed with an advanced SQCC decisive for the neoadjuvant treatment and a small separate adenocarcinoma of 1.5 cm diameter observed in the resection specimen.

### Tumor staging

Staging-parameters were re-evaluated for each case by two investigators (SB, PZ) accessing all available HE slides, pathology reports, and clinical files. As pleural invasion is pT relevant, it was re-assessed by re-evaluating the H&E slides and if necessary performing elastica-van-Gieson stains according to standard protocols [28].

All cases were re-staged according to TNM8 [18]. The T categories were included as size-adapted (respecting the proportion of residual tumor cells) and non-adapted variables. The adapted tumor size was reassessed according to the current IASLC recommendations by multiplying the vital tumor percentage with the described tumor bed extracted from the pathology report [16]. In case of missing H&E slides, sections were recut from the corresponding paraffin blocks.

Lymph-node stages were validated using the IASLC lymph node map [29]. Lymph nodes were free of residual tumor (ypN0) in 50/117 (42.7%) cases. Ipsilateral hilar lymph nodes were involved (ypN1) in 23/117 (19.7%) cases, ipsilateral mediastinal lymph nodes (ypN2) in 42/117 (35.9%) cases, and contralateral or supraclavicular lymph nodes (ypN3) in 2/117 (1.7%) cases. Five patients (4.3%) had distant metastases at the time of surgery.

### Tumor regression grade

Residual tumor content in the background of therapy-induced fibrosis and necrosis was evaluated by two investigators (SB, PZ) using a dual-head microscope, re-evaluating all available tumor material. In the few cases of discordance, the agreement was reached by consensus after thorough discussion. Two previous studies have reported a high reproducibility in the assessment of MPR in NSCLC [30, 31]. Specimen grossing as retrospectively evaluated for all tumors was for the majority of cases in line with the current IASLC guidelines advising complete inclusion of tumors <3 cm and sampling of at least 1 block per diameter of the tumor bed [16]. On average, 2.2 paraffin blocks were submitted per tumor bed diameter (median 2 [range 0.4–8.7]) as detailed in supplementary Table S-1. There were 14/117 (12%) borderline cases with marginally <1 block submitted per diameter tumor bed. Only 2 cases had ~ 0.5 a paraffin block or less submitted per tumor bed diameter, thus not adhering to the current recommendations. One case constituted a large carcinoma of 13 cm diameter with 5% residual tumor and the other one (80% residual tumor) was associated with considerable retrotumoral pneumonic remodeling which was not counted as tumor bed in our re-evaluation.

We assessed tumor regression as continuous variables using 1% increments till 10% and in 5% increments in cases showing >10% residual tumor in the primary tumor bed according to IASLC recommendations to calculate the adapted tumor size [16].

Regression was dichotomized into cases with and without MPR, currently defined as ≤10% residual tumor [15, 17]. In addition, histology-adapted MPR was determined for adenocarcinoma and non-adenocarcinoma cases separately, setting optimal cut-offs based on the proportion of residual tumor in the primary lesion to dichotomize survival using maximally selected rank statistic as described before [30].

**Fig. 1:**
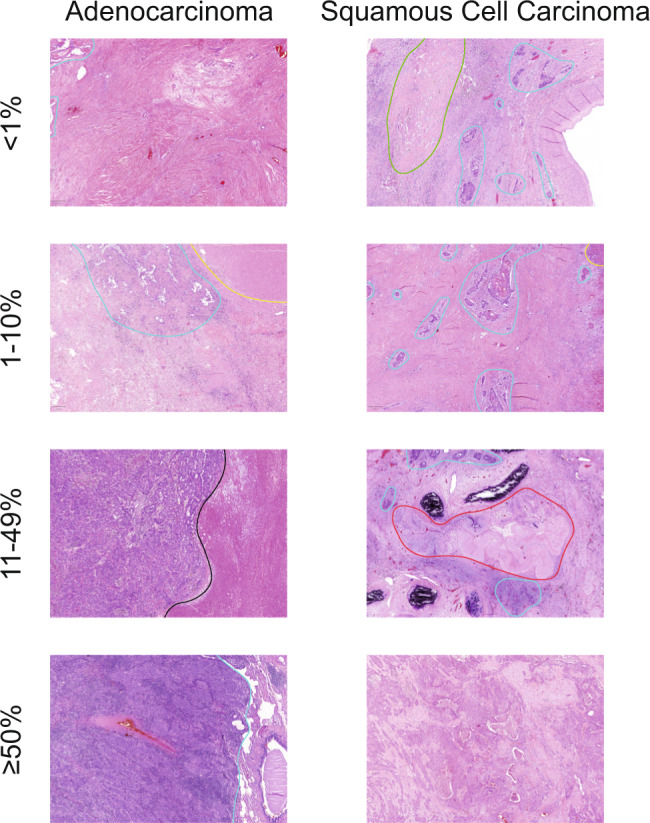
Representative H&E slides for estimation of the residual tumor. Light-blue: demarcation of tumor tissue, Yellow: demarcation of necrosis, Green: demarcation of cholesterol crystals, Red: demarcation of thickened/hyalinized vessels, Black: demarcation between tumor/necrosis.

Tumor regression grade (TRG) was assessed semi-quantitatively as the percentage of residual tumor cells in the primary tumor and regional lymph nodes using the following increments: no residual tumor (pCR), <1%, 1–10%, 11 to <50% and ≥50% residual tumor cells (Fig. 1) [14].

### Prognostic score

MPR, nodal infiltration (ypN), and the T-category (ypT-TNM8) were combined in a prognostic score (PRSC) similar to the scores described for gastric and oesophageal cancers after neoadjuvant treatment [32, 33]. Each factor was assigned a point value according to the respective prognostic impact (0 or 1 points each), the sum of which resulted in stratification into three risk categories: low risk 0–1 point, intermediate-risk 2 points, high risk 3 points (Table 2, Supplementary Fig. S-3). In line with our results for histology-adapted MPR (see below), we investigated the PRSC using the MPR cut-off of 10% and a histology-adapted PRSC adapting for different MPR cut-offs, using 10% in non-adenocarcinomas and 65% in adenocarcinomas.

**Table 2:** Criteria and scoring system of the prognostic score.

	Non-adapted prognostic score		Adapted prognostic score	
Parameter	0 points	1 point	0 points	1 point
ypT-TNM8	≤ypT2a	>ypT2a	≤ypT2a	>ypT2a
ypN-TNM8	ypN0	ypN1-3	ypN0	ypN1-3
Residual Tumor	≤10%	>10%	≤10%^a^/≤65%^b^	>10%^a^/>65%^b^

Scoring: 0–1 points, low risk; 2 points, intermediate risk; 3 points, high risk.

*ypT-TNM8* ypT according TNM eighth edition, *ypN-TNM8* ypN according TNM eighth edition.

^a^Non-adenocarcinoma.

^b^Adenocarcinoma.

### Statistical analyses

All analyses were performed using R software (version 3.6.3, https://cran.r-project.org) with suitable packages. Association between variables was assessed using Fisher’s exact test for nominal variables and Mann–Witney-U, Kruskal–Wallis tests for ordinal variables. For the correlation between ordinal or numerical variables, we used the rank correlation according to spearman. The maximally selected rank statistics were used for the selection of suitable cut-offs applying log-rank scores as the test statistic and approximated *p*-values according to Hothorn and Lausen [34]. Kaplan–Meier plots were used to visualize survival data with the corresponding *p*-values according to the log-rank test. Cox proportional hazard models were used for univariate and multivariate analyses. Univariate predictors were included in the multivariate model at *p* ≤ 0.1. The Akaike information criterion (AIC) and Bayesian information criterion (BIC) were used to compare the goodness-of-fit between the different prognostic models. This method adjusts the −2 log-likelihood statistics for the number of parameters in the model and the number of observations used. Smaller AIC and BIC indicate superior model fit with the probability of a better fit being *p*_*i*_. The level of significance was set at a two-sided *p* = 0.05.

## Results

### Stage migration due to size adjustment for ypT

We evaluated the migration of ypT and tumor stage after adjusting for the residual tumor in the primary tumor bed (Supplementary Table S-2). In summary, 27/117 (23.1%) cases migrated to a lower ypT category (not including changes within ypT1). This resulted in an adapted TNM stage in only 16/117 (13.7%) cases (Supplementary Table S-3).

In detail, a total of 10/117 (8.5%) node-negative cases were downstaged to stage I A 1 (ypT1b [*n* = 4], ypT1c [*n* = 3], ypT2a [*n* = 2], ypT2b [*n* = 1] to ypT1a). Two (1.7%) node-negative cases were downstaged to stage I A 2 (ypT2a [*n* = 1] and ypT3 [*n* = 1] to ypT1b). One (0.9%) node-negative case was downstaged to stage I A 3 (ypT2a to ypT1c). Three (2.6%) node-negative cases changed to stage I B (ypT2b [*n* = 1], ypT3 [*n* = 1], ypT4 [*n* = 1] to ypT2a). One (0.9%) case with positive hilar lymph nodes was downstaged to stage II B (ypT3 to ypT1b). Two (1.7%) node-negative cases were downstaged to stage II B due to a change from ypT4 to ypT3. Two (1.7%) cases with positive mediastinal lymph nodes were downstaged to III A due to a change from ypT3 to ypT2b. Two (1.7%) cases with ypT4 were downstaged from stage III B to stage III A due to migration to ypT1b and ypT2b.

### Prognostic value of TNM8

**Fig. 2:**
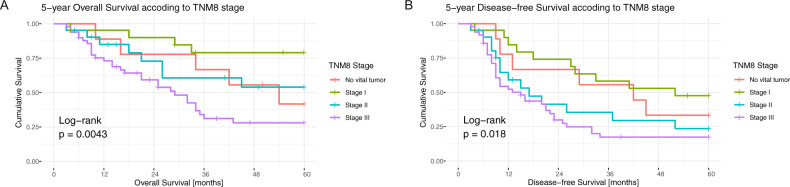
Survival according to TNM staging. Kaplan-Meier curves depicting 5-year overall survival (**A**) and 5-year disease-free survival (**B**) according to TNM (8th edition).

Both the adapted and non-adapted TNM8 were significantly associated with survival regarding the ypT-category or the tumor stage and OS or DFS. Figure 2 displays the survival curves for the non-adapted TNM8 (OS ypT *p* < 0.001 Fig. S-4A, stage *p* = 0.004 and DFS ypT *p* < 0.001 Fig. S-4C, stage *p* = 0.018). Supplementary Figs. 4B–D and 5 display the survival curves for the adapted TNM8 (OS ypT *p* < 0.001, stage *p* = 0.013 and DFS ypT *p* = 0.003, stage *p* = 0.025). The categories ypT2b and a pCR (=stage 0) had worse OS and DFS than the corresponding higher categories. This is most probably due to the small number of patients in these categories. Only three patients were staged ypT2b after size adjustment, two of which were downstaged from ypT3. Six patients with non-adapted ypT-categories were staged ypT2b, of whom two showed tumor progression after surgery and all others relapsed within 17 months after resection. Five of the nine cases with pCR died within the 5-year follow-up time, of which three patients relapsed and one patient was diagnosed with multiple myeloma 4 months prior to death. The AIC and BIC scores of the adapted TNM8 scoring system are higher compared to the original TNM8 indicating a worse fit (Table 3). However, the difference is only marginal and not sufficient to conclude a difference of performance between the models.

**Table 3:** Comparison of goodness-of-fit criteria between the applied prognostic models.

	TNM8	Adapted TNM8	MPR	Adapted MPR	PRSC	Adapted PRSC
df	3	3	1	1	2	2
OS						
AIC	382.4417	385.5961	385.7463	385.1203	372.9114	372.9499
BIC	387.9922	391.1465	387.5965	386.9705	376.6117	376.6502
DFS						
AIC	543.2423	544.0249	537.7455	538.2779	534.3239	532.7202
BIC	549.9009	550.6834	539.965	540.4974	538.7629	537.1592

*TNM8* stages according TNM 8th edition, *MPR* major pathological response, *PRSC* prognostic score, *df* degrees of freedom, *OS* overall survival, *DFS* disease-free survival, *AIC* Akaike information criterion, *BIC* Bayesian information criterion.

### Tumor regression

Assessing TRG, no residual tumor (defined as pCR) could be identified in 11/117 (9.4%) cases. Eighteen/117 (15.4%) cases showed <1%, 15/117 (12.8%) 1–10%, 16/117 (13.7%) 11–49% and 57/117 (48.7%) ≥50% residual tumor. pCR in the primary tumor bed was observed in 15 (12.8%) cases of which 4 cases had tumor-infiltrated lymph nodes with little pathologic regression considered to show in total <1% residual tumor. One case had a clinically diagnosed metastasis in the adrenal gland without available histological samples (regarded as pCR due to favorable follow-up data). Higher TRG correlated with lower TNM categories (ypT *p* < 0.001, ypN *p* < 0.001, stage *p* < 0.001 after exclusion of cases with pCR) and was associated with additional neoadjuvant radiotherapy (*p* < 0.001).

A median of 40% [IQR 0.5–80] residual tumor cells was detected in the primary lesion. For the entire cohort, we determined the most accurate cut-off for MPR at 10% residual tumor. A total of 44/117 (37.6%) cases presented ≤10% residual tumor, defining MPR. MPR correlated with lower TNM categories (ypT *p* < 0.001, ypN *p* < 0.001, stage *p* < 0.001 after exclusion of cases with pCR) and was associated with neoadjuvant radiochemotherapy (*p* < 0.001) and R0 status (*p* = 0.023).

### Tumor regression—histology-adapted percentage for MPR

In addition, we explored if divergent cut-offs would be preferable for different histological tumor types. The optimal cut-off was 65% residual tumor for adenocarcinomas and 9% residual tumor for SQCC. Histology adapted MPR was therefore set at ≤65% residual tumor for adenocarcinomas, representing 2/3, and ≤10% residual tumor for non-adenocarcinoma cases, resulting in 53/117 (45.3%) cases with adapted MPR. Adapted MPR correlated with lower TNM categories (ypT *p* < 0.001, ypN *p* < 0.001, stage *p* < 0.001 after exclusion of cases with pCR) and was associated with neoadjuvant radiochemotherapy (*p* = 0.001) and R0 status (*p* = 0.019).

### Prognostic value of tumor regression

Lower tumor regression was statistically significantly associated with shorter survival (OS *p* = 0.04 and DFS *p* = 0.02), with increasing HR per proportion of residual tumor for OS (HR 1.008 95% CI 1.000–1.015) and DFS (HR 1.007 95% CI 1.001–1.013). TRG was significantly associated with DFS (*p* = 0.006) but failed to show a significant association with OS (*p* = 0.091).

**Fig. 3:**
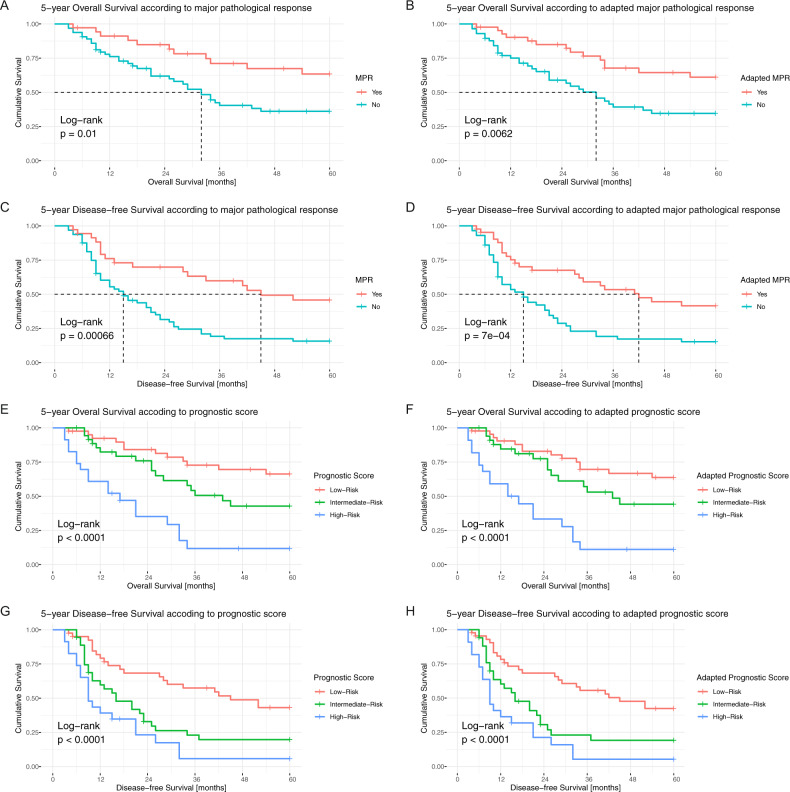
Survival according to tumor-response to neoadjuvant therapy assessed by different scoring systems. Kaplan–Meier curves depicting 5-year overall survival (**A**, **B**, **E**, **F**) and 5-year disease-free survival (**C**, **D**, **G**, **H**) according to non-adapted MPR (**A**)–(**C**) or prognostic score (**E**)–(**G**) and adapted MPR (**B**)–(**D**) or prognostic score (**F**–**H**).

A dichotomized evaluation using the 10% cut-off for MPR regardless of histological tumor type resulted in a statistically significant stratification for OS (*p* = 0.01, HR of 2.368 95% CI 1.203–4.663, Fig. 3A), and DFS (*p* < 0.001, HR 2.548 95% CI 1.461–4.443, Fig. 3C). In adenocarcinomas, adapted MPR had a significant impact on DFS (*p* = 0.027), but not on OS (*p* = 0.4, Supplementary Fig. S-6), presumably due to low sample size (*n* = 45). In non-adenocarcinoma cases, adapted MPR had a significant impact on OS (*p* = 0.01) and DFS (*p* = 0.013) with 55 cases included (Supplementary Fig. S-6). A dichotomized evaluation of the entire cohort using the adapted MPR had a highly significant impact on both OS (*p* = 0.006) and DFS (*p* < 0.001) and resulted in similar HR as when using the non-adapted MPR (Fig. 3B–D).

### Prognostic value of the prognostic score

The PRSC discriminated between three risk groups (Table 3): 48/117 (41%) patients were in the low-risk group, 41/117 (35%) patients in the intermediate and 28/117 (24%) in the high-risk group. PRSC risk groups showed an overall significant difference for OS (*p* < 0.001, Fig. 3E) with a significantly higher risk of death for patients in the high-risk group (HR 6.010 95% CI 2.8361–12.7400). For the low-risk group, the median OS was not reached. The intermediate-risk group had a median OS of 43 months (95% CI 28–NA) and the high-risk group a median OS of 17 months (95% CI 9–32). The low-risk group had a median DFS of 45 months (95% CI 28–NA), the intermediate-risk group a median DFS of 16 months (95% CI 10–25), and the high-risk group a median DFS of 9.5 months (95% CI 7–26). The overall difference in DFS was significant (*p* < 0.001, Fig. 3G), and patients at intermediate risk (HR 2.2110 95% CI 1.2410–3.9380) and high risk (HR 3.7940 95% CI 2.0180–7.1350) had a significantly higher risk for death or recurrence.

The histology adapted PRSC using adapted MPR cut-offs resulted in the migration of two adenocarcinomas from the intermediate to the low-risk group. Overall, the stratification had a statistically significant impact on survival (OS *p* < 0.001, DFS *p* < 0.001, Fig. 3F–H) and the HR was comparable to the non-adapted PRSC.

### Multivariate analyses

Multivariate analyses were conducted separately for MPR/adapted MPR, TNM8 stages/adapted stages, and the PRSC/adapted PRSC as these parameters were significantly associated with each other. Table 4 represents the final multivariate models of non-adapted prognostic markers including only parameters without any association to stage, MPR or PRSC.

**Table 4:** Multivariate analyses of the non-adapted major prognostic systems for overall survival.

	Multivariate—Model TNM8				Multivariate—Model MPR				Multivariate—Model PRSC			
	*N* = 90 (%)	HR	95% CI	*p*	*N* = 99 (%)	HR	95% CI	*p*	*N* = 99 (%)	HR	95% CI	*p*
Stage TNM8 (vs. Stage I)												
Stage II	20 (22.2)	2.406	0.703–8.234	0.162		–	–	–		–	–	–
Stage III	**50 (55.6)**	**4.956**	**1.735–14.157**	**0.003**		–	–	–		–	–	–
No-MPR (vs. MPR)		–	–	–	**64 (64.6)**	**2.432**	**1.192–4.962**	**0.015**		–	–	–
PRSC (vs. low)												
Intermediate		–	–	–		–	–	–	36 (36.4)	2.082	0.9623–4.506	0.063
High		–	–	–		–	–	–	**23 (23.2)**	**5.692**	**2.527–12.820**	**<0.001**
Regimen change (vs. no-change)	**18 (20)**	**2.390**	**1.210–4.718**	**0.012**	**20 (20.2)**	**2.597**	**1.350–4.996**	**0.004**	**20 (20.2)**	**2.363**	**1.223–4.557**	**0.010**
Surgery (vs. Lobectomy)												
Bilobectomy		*	*	*	5 (5.1)	1.282	0.303–5.429	0.736	5 (5.1)	1.010	0.236–4.322	0.989
Pneumonectomy		*	*	*	35 (35.4)	1.828	0.993–3.367	0.053	35 (35.4)	1.383	0.735–2.605	0.315

Bold values indicate statistical significance *p* < 0.05

*TNM8* stage according TNM eighth edition, *HR* hazard ratio, *95% CI* 95% confidence interval, *MPR* major pathological response, *PRSC* prognostic score.

Non-adapted and adapted MPR were significant prognostic predictors for OS independently of a change in neoadjuvant chemotherapy or conducted surgical procedure.

Due to the small sample size of cases with pCR, only stages I–III were included in the multivariate model. Stage III patients of the non-adapted and adapted TNM8 staging system had a significantly higher risk for death irrespective of a change in neoadjuvant chemotherapy.

For the non-adapted PRSC, patients of the high-risk group had worse OS independent of a change in neoadjuvant chemotherapy or conducted surgical procedure. For the adapted PRSC, patients of the intermediate- or high-risk group had worse OS independent of a change in neoadjuvant chemotherapy.

### Comparison between the different prognostic models

The PRSC model, both using the histology adapted cut-offs and the 10% cut-off regardless of histology, was superior compared to the stratification using MPR alone or the TNM8 systems, visualized by lower AIC and BIC values (Table 3). The probability of the non-adapted TNM8 system to better predict OS (*p*_TNM_ = 0.8%) or DFS (*p*_TNM_ = 1.2%) compared to the PRSC was below 5%.

## Discussion

The present study compares the prognostic significance of the TNM8 system, tumor regression grading and a combined PRSC in a clinicopathological well-annotated single-center retrospective cohort of NSCLC resected after neoadjuvant treatment.

The UICC/AJCC TNM classification remains the guideline of choice to predict prognosis in patients with NSCLC. However, most of the validation studies explicitly excluded patients with neoadjuvant treatment regimens, and additional studies are needed to apply and confirm the IASLC-recommended yT-adjustment after neoadjuvant therapy [16, 19–21]. In our cohort, the integration of regression in the yT-category alone did not yield a better fitting prognostic model compared to the original TNM8 system. Only 16 (13.7%) cases were downstaged due to adaptation of tumor size according to the current IASLC recommendations [16]. This could be explained by a high proportion of cases with ≥50% of residual tumor after neoadjuvant therapy in our cohort (*n* = 57, 48.7%).

We confirm the prognostic significance of MPR in NSCLC. These results are in line with existing literature propagating MPR as a possible surrogate endpoint in neoadjuvant trials [3, 15, 17]. In concordance with Qu and colleagues, we support the assessment of residual tumor in steps of 5–10% and confirm 65% as the optimal cut-off for assessing MPR in adenocarcinomas. Our findings support the adaptation of MPR according to the histologic tumor type [30, 35, 36].

Compared to the classic TNM8 system, the MPR model performed comparable regarding OS but was superior regarding DFS. The strong association of MPR and additional neoadjuvant radiotherapy may provide a possible explanation. In most of the previous reports on tumor regression after neoadjuvant radiochemotherapy, the fraction of cases achieving MPR was higher compared to studies including only patients after neoadjuvant chemotherapy [13, 35, 37–41]. Among our cases eligible for survival analyses, only 36 (36%) showed MPR corresponding to 64.3% of the group receiving neoadjuvant radiochemotherapy and 26.5% of the group receiving neoadjuvant chemotherapy. Neoadjuvant radiochemotherapy seems to result in better loco-regional control of the tumor and higher rates of complete resection, thus prolonging the time to recurrent disease [3, 42].

The combination of multiple parameters into a prognostic score in order to better stratify patients has already been suggested for locally advanced primary resected NSCLC [8, 9]. Liang et al. established a prognostic nomogram combining age, histology, number of sampled lymph nodes, gender, T-category, and N-category predicting OS more accurately than the TNM staging system [9]. Pilotto et al. developed a score for SQCC combining age, T-category, nodal status, and tumor grading [8]. This group was able to validate their model in a multicentric retrospective cohort and showed the prognostic impact of adjuvant or neoadjuvant therapy in the intermediate and high-risk groups using propensity score analyses [10]. However, including tumor grading into prognostic scores is problematic, as (a) grading should not be assessed after neoadjuvant therapy due to therapy-induced morphological changes and (b) despite widely used propositions e.g., for adenocarcinoma of the lung, there are to date no universally accepted grading systems for NSCLC [27]. In addition, a simpler model for routine clinical application is necessary. For gastric and oesophageal cancers, such combined scores have demonstrated a superior accuracy compared to the classic TNM staging system, but to our knowledge, no such combined scores exist for NSCLC after neoadjuvant treatment [32, 33]. We constructed a prognostic score integrating the same components: T-category, nodal status, and MPR. This model showed superior prognostic accuracy over the TNM staging system even after adapting for tumor size (T-category) according to residual tumor in the primary tumor bed.

The strength of our study is the complete clinicopathological re-validation of the cohort including homogenization of stage and tumor type according to current guidelines and integration of the recommendations of the IASLC, thereby including information on tumor regression in the TNM stages [16]. The limitation of our study is inherent in the study design using a real-life cohort, thus the therapeutic regimens had been adapted to the needs of the patients. This becomes obvious regarding the vast range of administered cycles of chemotherapy and the varying time intervals between neoadjuvant therapy and surgery. However, the time intervals had no significant prognostic impact as assessed by univariate cox-regression analysis. Using a real-life cohort resected during a large time-frame of 16 years resulted also in the lack of a standardized grossing procedure. Even though, we deem our results have not been substantially biased. Only 2 out of 117 cases were insufficiently sampled according to current IASLC guidelines [16]. Both cases were squamous cell carcinomas and showed 80% respectively 5% residual tumor in the primary lesion.

Our sample size is comparable to other retrospective cohorts investigating potential survival predictors in NSCLC after neoadjuvant treatment and the role of tumor regression: Pöttgen et al. (*n* = 157), Remark et al. (*n* = 122), Betticher et al. (*n* = 75) or Pataer et al. (*n* = 192) [13, 15, 37, 43].

In conclusion, we were able to demonstrate the prognostic validity of the TNM8 classification, both using sizes adapted and non-adapted T-category, in a cohort of curatively resected NSCLC after neoadjuvant treatment. Since the prognosticating performance was considerably increased by integrating the non-adapted ypT, nodal status, and MPR into a combined prognostic score, pathologic tumor response should be acknowledged as an additional relevant prognostic factor in NSCLC treated by neoadjuvant therapy.

## Supplementary information

Supplementary Word Document

Supplementary Table S-1

Supplementary Table S-4

Supplementary Table S-5

Supplementary Script S-1

## Data Availability

Supplementary Table S-4 and S-5: Detailed data on the included patients. Supplementary Script S-1: Script used for cut-off determination and survival analyses in R.
