# Supplementary material for: A prognostic score for non-small cell lung cancer resected after neoadjuvant therapy in comparison with the tumor-node-metastases classification and major pathological response
Source: Mod Pathol. 2021 Mar 13;34(7):1333–44. doi: 10.1038/s41379-021-00777-y (PMC8216907; doi:10.1038/s41379-021-00777-y)
Supplement: Supplementary file 1 — Supplementary Word Document [file 41379_2021_777_MOESM1_ESM.docx]

**SUPPLEMENTARY MATERIAL**

**Contents:**

*Supplementary Figures*

| Figure S-1 | Flow chart depicting patient exclusion and causes for exclusion | Page 2 |
| --- | --- | --- |
| Figure S-2 | Overall survival and disease-free survival of the cohort | Page 3 |
| Figure S-3 | Determination of the optimal cut-off for ypT and ypN and Kaplan-Meier plots applying these cut-offs | Page 4 |
| Figure S-4 | Kaplan-Meier curves depicting 5-year overall survival (A-B) and 5-year disease-free survival (C-D) according to ypT-categories (A-C) and size-adapted ypT-categories (B-D) | Page 5 |
| Figure S-5 | Kaplan-Meier curves depicting 5-year overall survival (A) and 5-year disease-free survival (B) according to size-adapted stages | Page 6 |
| Figure S-6 | Determination of the optimal cut-off for major pathological response according to underlying histology using maximally selected rank statistic and corresponding Kaplan-Meier plots | Page 7 |

*Supplementary Tables*

| Table S-2 | Cross tabulation for comparison of non-adapted and size-adapted ypT categories | Page 8 |
| --- | --- | --- |
| Table S-3 | Cross tabulation for comparison of non-adapted and size-adapted stages | Page 9 |

**
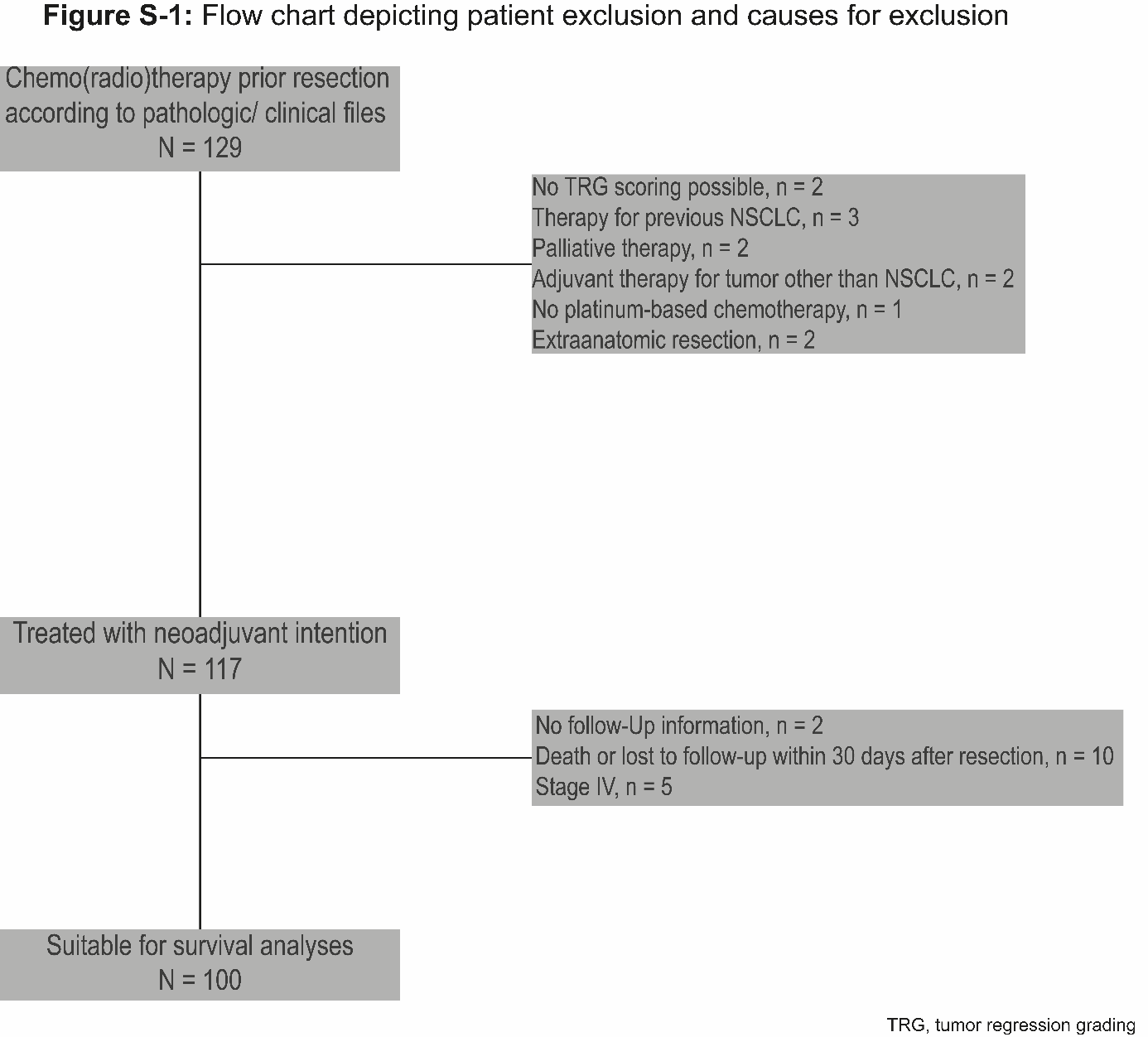
**

**
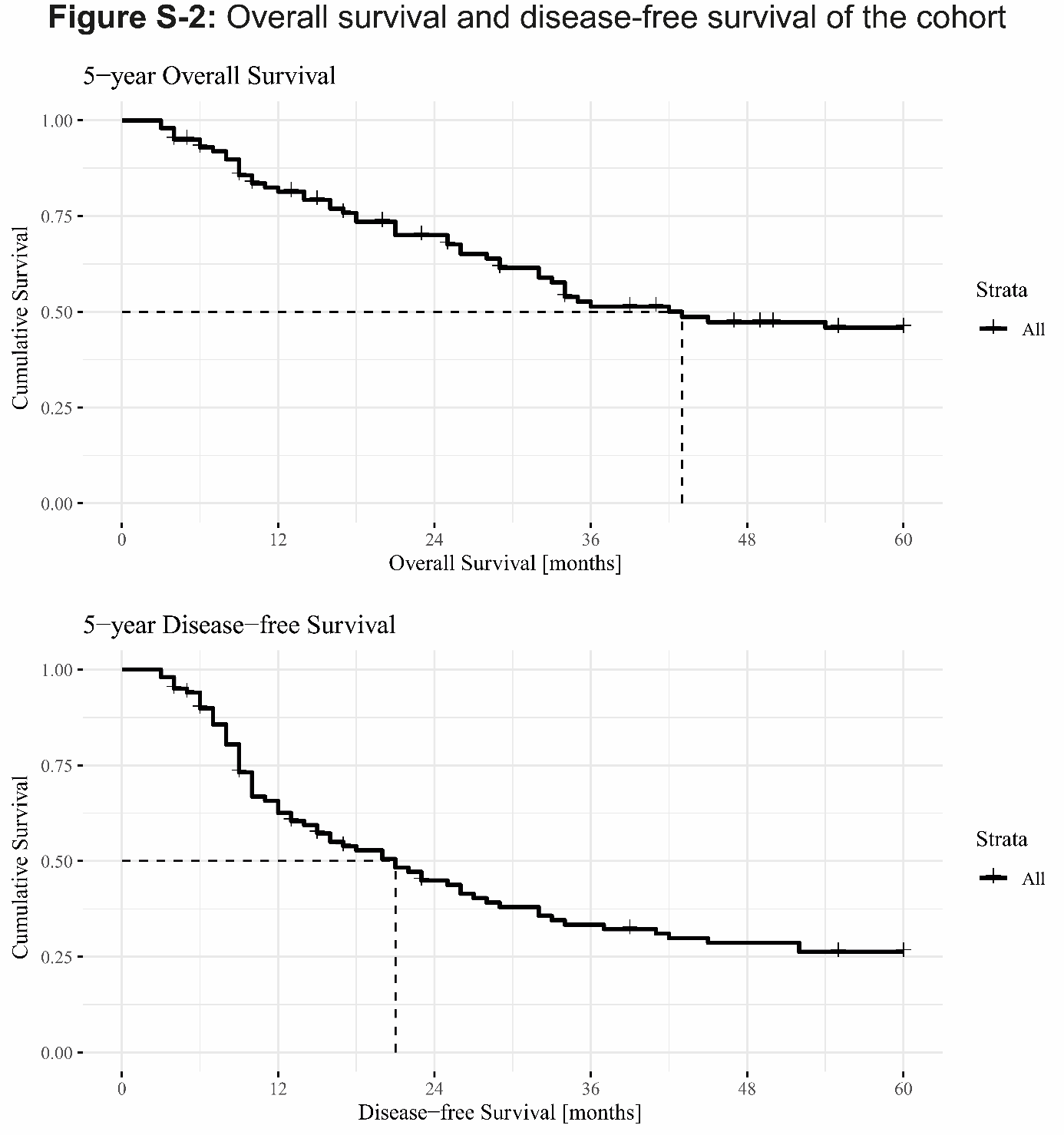
**

**
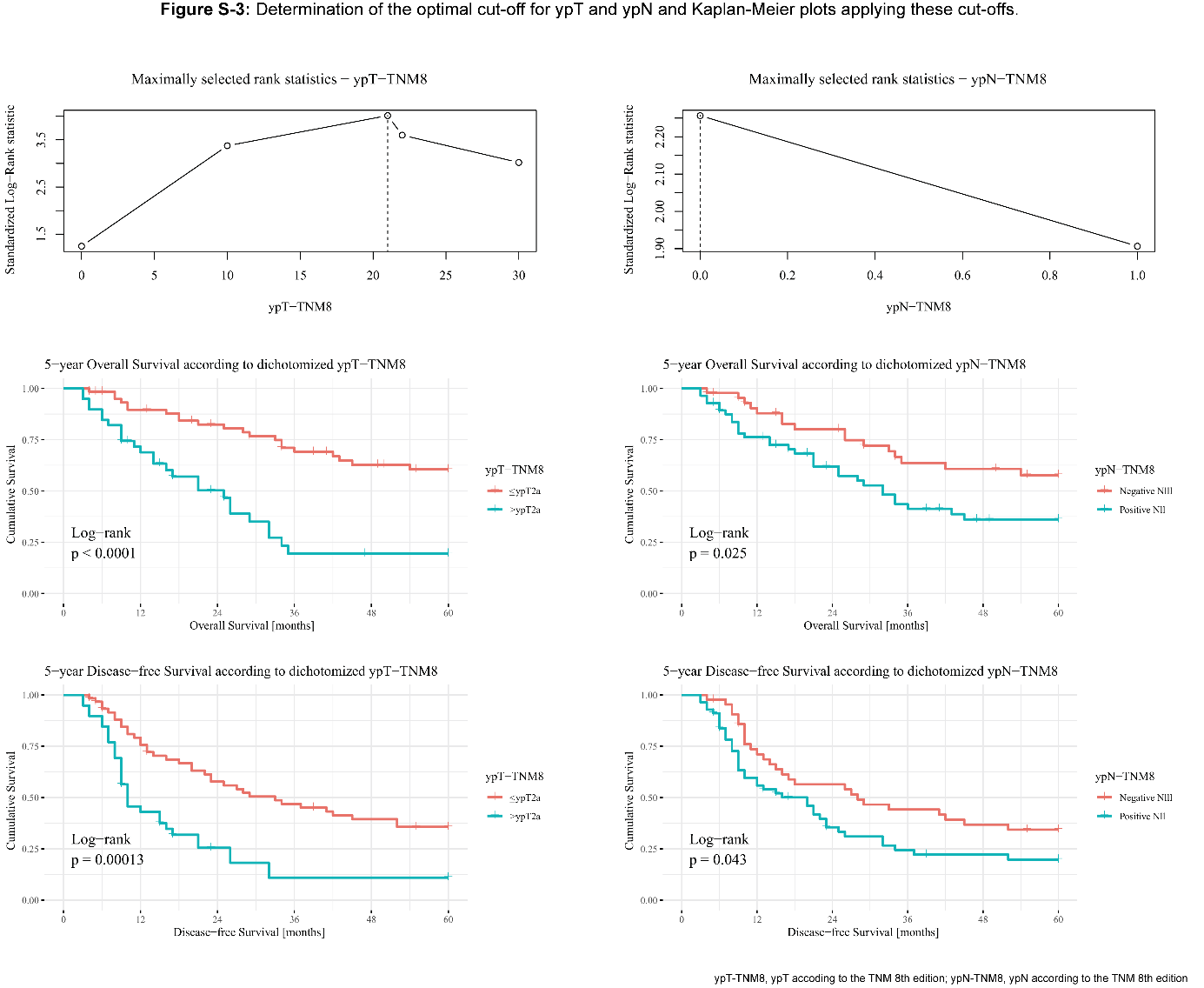
**

**
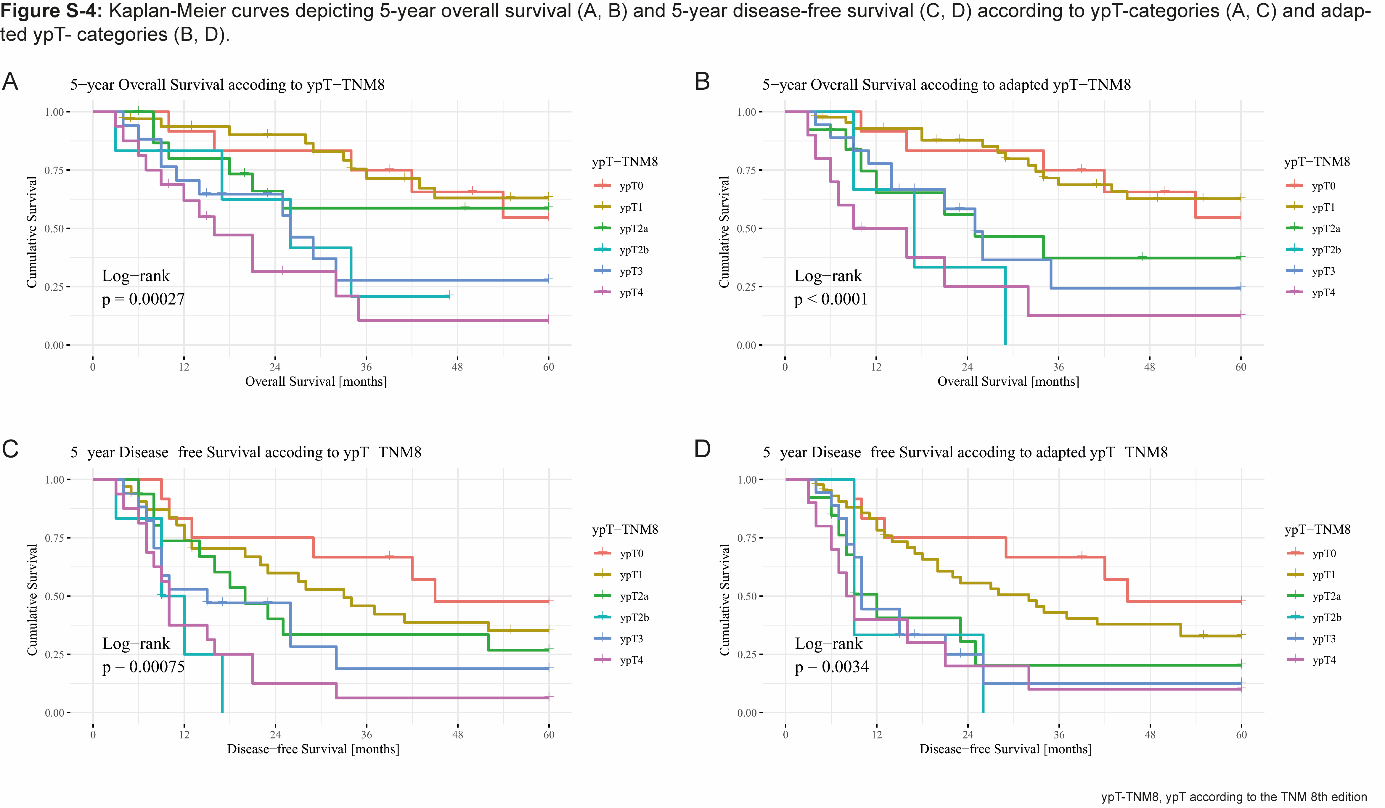
**

**
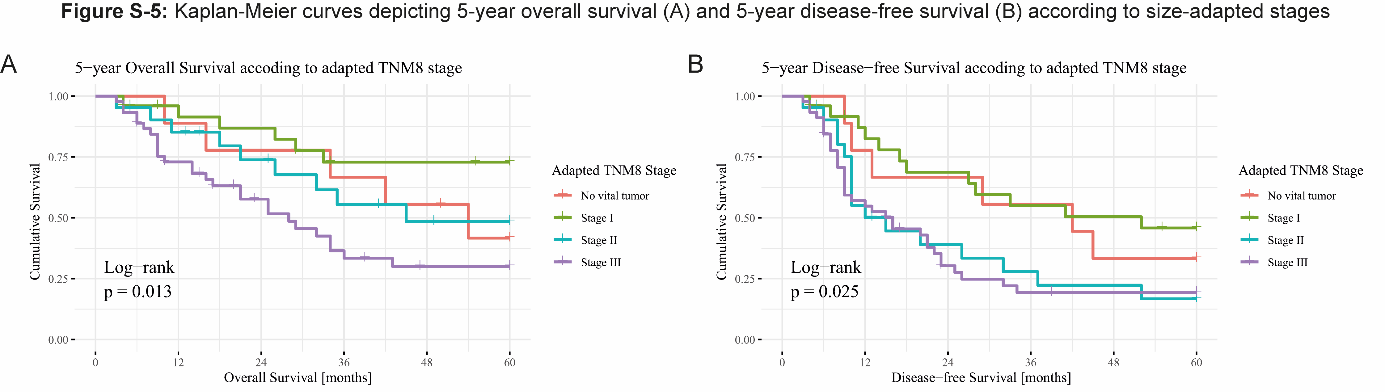
**

**
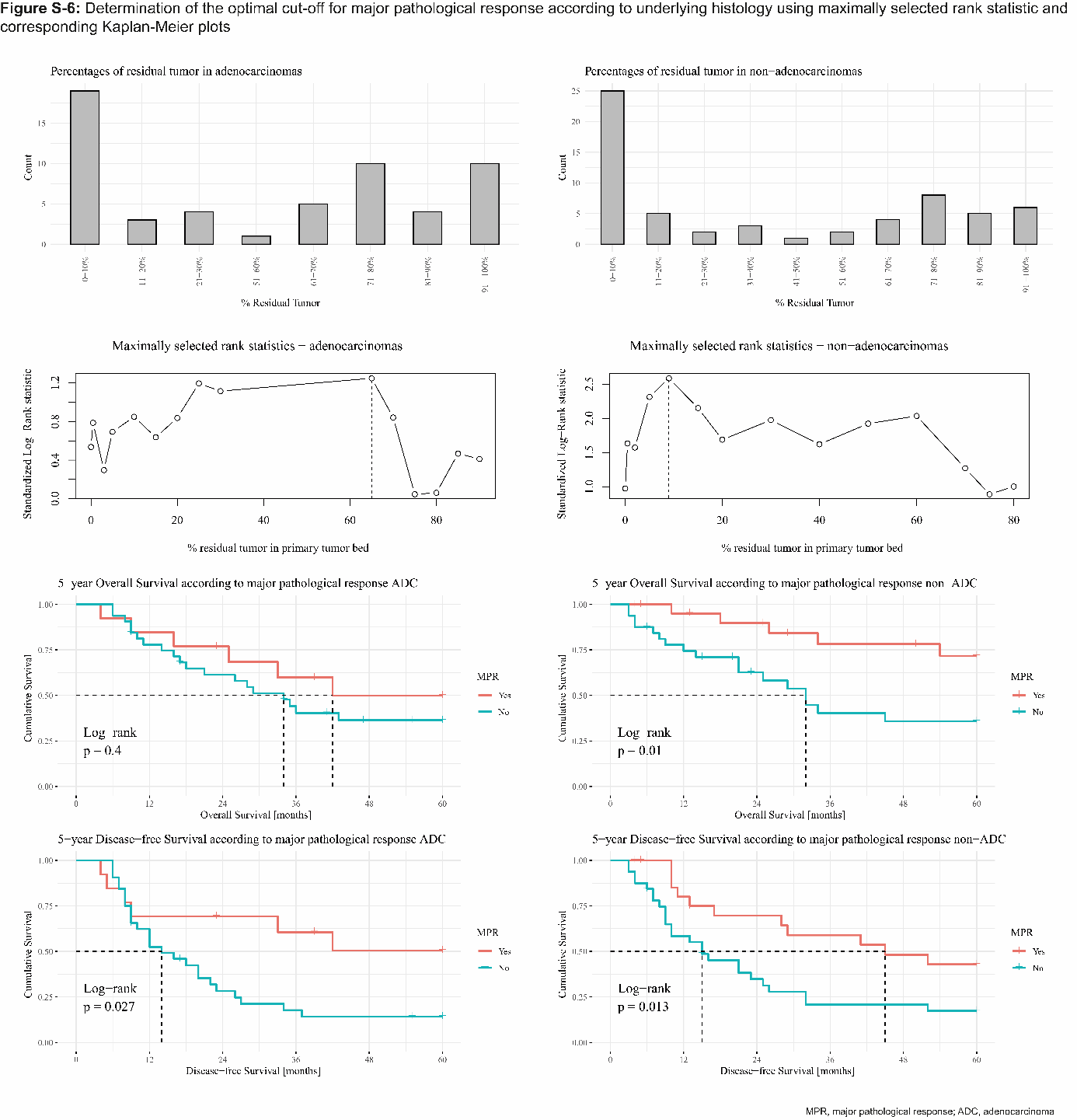
**

Table S-2: Cross tabulation for comparison of non-adapted and size-adapted ypT categories

|  |  | Non-adapted ypT | | | | | | |
| --- | --- | --- | --- | --- | --- | --- | --- | --- |
|  |  | ypT0 | ypT1 | ypT2a | ypT2b | ypT3 | ypT4 | Total |
| Size-adapted ypT | ypT0 | 15 |  |  |  |  |  | 15 |
|  | ypT1 |  | 38 | 8 | 1 | 2 | 1 | 50 |
|  | ypT2a |  |  | 9 | 4 | 1 | 1 | 15 |
|  | ypT2b |  |  |  | 1 | 2 | 1 | 4 |
|  | ypT3 |  |  |  |  | 15 | 6 | 21 |
|  | ypT4 |  |  |  |  |  | 12 | 12 |
|  | Total | 15 | 33 | 20 | 7 | 20 | 22 | 117 |

TNM8, TNM 8th edition

Table S-3: Cross tabulation for comparison of non-adapted and size-adapted stages

|  |  | Non-adapted TNM8 stage | | | | | | | | | |
| --- | --- | --- | --- | --- | --- | --- | --- | --- | --- | --- | --- |
|  |  | «0» | IA | IB | IIA | IIB | IIIA | IIIB | IIIC | IV | Total |
| Adapted TNM8 stage | «0» | 10 |  |  |  |  |  |  |  |  | 10 |
|  | IA |  | 19 | 4 | 1 | 1 |  |  |  |  | 25 |
|  | IB |  |  |  | 1 | 1 | 1 |  |  |  | 3 |
|  | IIA |  |  |  |  |  |  |  |  |  | 0 |
|  | IIB |  |  |  |  | 21 | 3 |  |  |  | 24 |
|  | IIIA |  |  |  |  |  | 31 | 4 |  |  | 35 |
|  | IIIB |  |  |  |  |  |  | 14 |  |  | 14 |
|  | IIIC |  |  |  |  |  |  |  | 1 |  | 1 |
|  | IV |  |  |  |  |  |  |  |  | 5 | 5 |
|  | Total | 10 | 16 | 6 | 2 | 23 | 36 | 18 | 1 | 5 | 117 |

TNM8, TNM 8th edition
